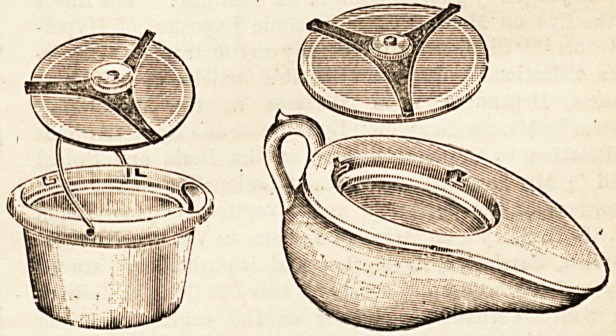# New Appliances and Things Medical

**Published:** 1900-04-14

**Authors:** 


### NEW APPLIANCES AND THINGS MEDICAL.

[We shall be glad to receive, at our Office, 28 & 29, Southampton Street Strand, London, W.C., from the manufacturers, specimens of all new preparations and appliances which may be brought out from time to time.]

### THE "KILLGERM" PATENT TYPHOID BUCKET AND BED PAN.

(The Killgerm Company Limited, Cleckheaton, Yorks.)

This patent arrangement, the nature of which is best explained by the accomp nying woodcuts' is one which is sure to prove of the greatest utility in all cases in which it is desired for any reason to preserve the evacuations, either for purposes of disinfection or in order that they may be inspected by the doctor. Even where a bed pan has to be carried through a ward or a passage it is a great advantage to have it tightly covered and made secure from all possibility of splashing or emitting odours, as is accomplished at once and in the simplest manner by the invention before us.

## Figures and Tables

**Figure f1:**